# Unsupervised Characterization of Prediction Error Markers in Unisensory and Multisensory Streams Reveal the Spatiotemporal Hierarchy of Cortical Information Processing

**DOI:** 10.1523/ENEURO.0251-23.2024

**Published:** 2024-05-02

**Authors:** Priyanka Ghosh, Siddharth Talwar, Arpan Banerjee

**Affiliations:** Cognitive Brain Dynamics Lab, National Brain Research Centre, Gurgaon 122052, India

**Keywords:** MMN, multisensory facilitation, P300, prediction error, source localization

## Abstract

Elicited upon violation of regularity in stimulus presentation, mismatch negativity (MMN) reflects the brain's ability to perform automatic comparisons between consecutive stimuli and provides an electrophysiological index of sensory error detection whereas P300 is associated with cognitive processes such as updating of the working memory. To date, there has been extensive research on the roles of MMN and P300 individually, because of their potential to be used as clinical markers of consciousness and attention, respectively. Here, we intend to explore with an unsupervised and rigorous source estimation approach, the underlying cortical generators of MMN and P300, in the context of prediction error propagation along the hierarchies of brain information processing in healthy human participants. The existing methods of characterizing the two ERPs involve only approximate estimations of their amplitudes and latencies based on specific sensors of interest. Our objective is twofold: first, we introduce a novel data-driven unsupervised approach to compute latencies and amplitude of ERP components accurately on an individual-subject basis and reconfirm earlier findings. Second, we demonstrate that in multisensory environments, MMN generators seem to reflect a significant overlap of “modality-specific” and “modality-independent” information processing while P300 generators mark a shift toward completely “modality-independent” processing. Advancing earlier understanding that multisensory contexts speed up early sensory processing, our study reveals that temporal facilitation extends to even the later components of prediction error processing, using EEG experiments. Such knowledge can be of value to clinical research for characterizing the key developmental stages of lifespan aging, schizophrenia, and depression.

## Significance Statement

Widely studied ERPs—MMN and P300—have been considered in the literature to be neural markers of prediction error propagation. Here, we use a data-driven unsupervised method to identify the amplitude and latencies of MMN and P300 from EEG-ERP signals and accurately estimate their underlying cortical sources, in auditory, visual, and audiovisual contexts. Subsequently, we could demonstrate that the multisensory/audiovisual contexts speed up the processing times of prediction mismatch compared with unisensory scenarios, and modality-specific processing at the MMN stage gives way to modality-independent processing at the P300 stage, thus capturing the hierarchy of prediction error processing. The results confirm previously speculated understanding of the cortical processing hierarchies often found in the literature of perceptual and cognitive processes.

## Introduction

Prediction error propagation along sensory streams is conceptualized to be organized hierarchically (Friston, 2005), consequently updating the internal model of the world at each hierarchical stage (Wacongne et al., 2011). Prediction mismatches are perceived through multiple sensory modalities (Di Luca et al., 2009); however, the internal mental models of sight, sound, smell, taste, and touch are hypothesized to be integrated with our existing cognitive schemata (Talsma, 2015). On the other hand, studies have provided evidence that multisensory integration can also happen in parallel and, at times speed up the speed of processing (Molholm et al., 2002; Van Wassenhove et al., 2005; Dhamala et al., 2007). This raises the question—what are the time scales corresponding to modality-specific and modality-independent processing during prediction mismatch? The answer can unravel the fundamental neural operations driving the processing of prediction errors regardless of a specific sensory stream. The first stage of multisensory integration was identified ∼100 ms of speech stimulus onset, from the appearance of a faster audiovisual N100—a robust ERP component, compared to the unisensory auditory analog (Van Wassenhove et al., 2005; Stekelenburg and Vroomen, 2007). Such evidences of multisensory facilitation are, however, not established for the later stages of information processing. The overarching goal of this article is to use the ERPs from human electroencephalogram (EEG) recordings in tandem with unsupervised signal processing approaches to index the temporal markers of prediction errors and their underlying cortical sources across different sensory modalities.

Prediction errors are indexed by the mismatch negativity (MMN) and the P300 components in the literature (Chennu et al., 2013; Stefanics et al., 2014; Calcus et al., 2015; Aitchison and Lengyel, 2017; Banellis et al., 2020). MMN is a negative deflection ∼100–250 ms poststimulus onset, in the difference waveform between deviant and standard EEG signals (Näätänen et al., 1978, 2007; Näätänen and Michie, 1979; Sams et al., 1985; Garrido et al., 2009). P300 is a positive deflection with a latency between 250 and 500 ms (Sutton et al., 1967; Giard et al., 1990; Polich, 2007). The key difference is that MMN is known to index any deviance in the absence of consciousness (Atienza et al., 2001; Koelsch et al., 2006) whereas P300 indexes possible attention switches and, hence, a conscious perception of stimulus change (Sutton et al., 1967; Giard et al., 1990; Polich, 2007). The latency of audiovisual MMN (avMMN) resembled closely to the latency of audio MMN (and not visual MMN; Shiramatsu et al., 2021), but a previous study (Sittiprapaporn, 2012) reported shorter visual and audiovisual MMN latencies than auditory MMN. Similarly, others have reported a significant early onset of P300 latency (Stefanics et al., 2005; Marucci et al., 2021) as well as increased amplitude (Fleming et al., 2020; Marucci et al., 2021) during multimodal stimulation (visual-audio-tactile and visual-audio) as compared to corresponding unimodal stimulations, but another study by Feng et al. (2008) did not observe such multisensory benefits for P300 latencies. Thus, latency and amplitude of MMN/P300 vary with experimental parameters, for example, stimulus duration, occurrence probability, and discrimination difficulty (Magliero et al., 1984; Patel and Azzam, 2005; Polich, 2007) as well as physiological variables, for example, age and attention (Polich et al., 1990; Blackwood, 2000; Dinteren et al., 2014; Erickson et al., 2016) leading to such disparate findings.

In this article, we hypothesize (1) the faster responses to multisensory stimuli can be seen in the middle/late processing stages of prediction errors, that is, for MMN/P300, and (2) there exists a modality-specific sensory-cognitive dissociation in the source distribution of MMN and P300. Previous MMN and P300 studies have been limited in their use of averaging methods across electrodes or of choosing just the conventional central electrodes (e.g., Fz, Cz, and Pz; Gonsalvez and Polich, 2002; Gonsalvez et al., 2007), which may not effectively capture the true MMN and P300 latencies in the first place. Additionally, cross-sensory modality, where the modality of the frequent and the deviant stimuli are different, may recruit different brain regions impacting the processing speeds of deviant stimuli. To resolve these issues, we employed an unsupervised dimensionality reduction technique across the sensor space to create comparable filters on which sensor data from different conditions, unisensory, multisensory, and cross-modal, can be projected for characterization of the ERP latencies/amplitudes across different sensory modalities. This novel approach to determining latencies provides an accurate estimation of modality-specific changes in the prediction error markers. Furthermore, we perform rigorous source analysis using coregistration with individual subject's MRI data to reveal the overlapping cortical networks and specialized brain regions specific to unimodal, multimodal, and cross-modal MMN and P300.

## Materials and Methods

### Ethics statement

The study was carried out following the ethical guidelines and prior approval of the Institutional Human Ethics Committee (IHEC) of the National Brain Research Centre, India. Written informed consent was obtained from all participants before the commencement of the experiment, and they were remunerated for the time of their participation.

### Participants

Twenty-two healthy volunteers (nine males and 13 females) in the age group of 22–43 (mean = 25.7, SD = ±4.19) years participated in the study. They had normal or corrected-to-normal vision and were right-handed. All participants had university degrees or higher and reported no history of neurological or audiological problems. They were requested to avoid the intake of any medication/stimulant (e.g., sedative and coffee) before the experiment.

### Stimuli

The experiment consisted of five different conditions and each condition consisted of two categories of stimuli, i.e., repetitive/frequent/standard and non-repetitive/oddball/deviant. Two of the five conditions presented were unimodal, i.e., audio only and visual only; the third was bimodal, i.e., audiovisual; and the remaining two were cross-modal in nature. In the first three conditions, the standard and the oddball stimuli were of the same sensory modality, which means that the audio-only condition comprised an audio standard and an audio oddball stimulus; the visual-only condition comprised a visual standard and a visual oddball stimulus; and the audiovisual (AV) condition comprised of an audiovisual standard and an audiovisual oddball stimulus. The remaining two conditions, namely, the cross-audio consisted of an audio deviant and a visual standard; and the cross-visual consisted of a visual oddball and an audio repetitive stimulus. The contents of each condition are tabulated in Table 1. Each condition consisted of 400 trials, out of which oddballs constituted 14% of the total trials. Each condition was presented in the form of a block of 100 stimuli, where the standard and oddball stimuli of a particular condition were presented in random order and the number of oddball stimuli varied across each block. There were 20 such blocks (five conditions × 4 blocks) that were randomized and presented to the participants. The participants were asked to count the number of oddballs presented in each block and report at the end of the block to ensure they attended to the whole stimulus (the results of counting accuracy for each participant are presented as Extended Data Table 1-1).

**Table 1.** The table lists the standard and the deviant stimuli used in our oddball paradigm for all the five sensory modality conditions

Condition	Standard/frequent/repetitive	Deviant/oddball/infrequent/nonrepetitive
Unimodal audio (A)	261.6 Hz	523.3 Hz
Unimodal visual (V)	Blue square	Red triangle
Bimodal audiovisual (AV)	261.6 Hz and blue square	523.3 Hz and red triangle
Cross-modal (audio deviant CrA)	Blue square	523.3 Hz
Cross-modal (visual deviant CrV)	261.6 Hz	Red triangle

Extended Data Table 1-1 has details on the accuracy of participants.

10.1523/ENEURO.0251-23.2024.t1-1 Table 1-1 Counting accuracy of participants in different task conditions. Download Table 1-1, DOCX file.

The stimuli of the visual-only condition consisted of a standard blue square and a deviant red triangle. The auditory stimuli were inspired by musical notes, the standard as the C4 note and the deviant as the C5 note (higher octave), according to the tuning of the A440 pitch standard. All stimuli were presented on a white background on a 21″ LED screen (1,280 × 1,024 pixels). The participants were asked to keep their eyes open and fixate on a central cross on the screen during the presentation of all auditory stimuli. The inter-stimulus interval also consisted of the same cross-fixation. The length of each oddball and standard stimulus was 350 ms, and the inter-trial interval ranged between 800 and 1,200 ms (mean = 1,000 ms). AV and cross-modal conditions were constructed from combinations of the audio-only and the visual-only stimuli (listed in Table 1).

### Data acquisition

EEG was recorded with 64 Ag/AgCl electrodes, using a Neuroscan System (Compumedics NeuroScan, SynAmps2). The electrodes were attached to an elastic cap in a 10–20 international system montage. The data were acquired at a sampling rate of 1,000 Hz with the default reference near Cz, grounded to electrode AFz. During the experiment, the participants were seated comfortably at a distance of 60–70 cm from the monitor in a sound-attenuated and dimly lit room. The participants were requested to make minimal body movements and blink normally during the experiment. The impedance of all electrodes was initially set below 5 kΩ and was also monitored in between blocks. Additionally, head digitization was performed using Polhemus Fastrak (Polhemus) to mark the position of the electrodes and the fiducials based on the placement of the cap on individual participants at the end of the entire EEG session. Individual T1-weighted structural images (MPRAGE) were also obtained using a 3.0T Philips Achieva MRI scanner (TR = 8.4 ms, FOV = 250 × 230 × 170, flip angle = 8°).

### Preprocessing

EEG was acquired from 22 participants out of which data from 1 participant was discarded due to noisy recordings. The raw data of the remaining 21 participants were imported, and each block was epoched. A high-pass filter of 0.1 Hz was applied to the data to remove slow drifts in the signal. The data were visually inspected further, and one channel (F4 in three subjects) was interpolated to neighboring electrodes. To identify and remove blink artifacts from the data, independent component analysis (ICA) was employed for each block. Only the blink component obtained as the independent component (IC) from eye regions was visually identified and subsequently, that IC was rejected. Furthermore, all blocks were subjected to a low-pass filter of <45 Hz and then, each block was epoched to trials of [−500 550] ms where 0 ms marked the onset of the stimulus. The trials were further divided into standard and oddball categories, baseline correction was applied using the prestimulus activity and the data was rereferenced to linked mastoids. Trials with signal amplitude >100 µV and lesser than −100 µV were removed and at most 6% of all oddball trials were discarded per subject. Then we equalized the number of oddball and standard trials within each separate condition for each participant by randomly selecting standard trials. The following are the total number of oddballs or standards in each condition with mean and standard deviation across participants—audio-only condition was 1,416 (mean = 67.4286, SD = 5.835), visual-only was 1,397 (mean = 66.5238, SD = 8.2257), AV was 1,450 (mean = 69.0476, SD = 2.8544), cross-audio was 1,468 (mean = 69.9048, SD = 1.5781), and cross-visual was 1,461 (mean = 69.5714, SD = 1.1650). All analyses were done using the MATLAB-based FieldTrip toolbox developed at Donders Institute for Brain, Cognition and Behaviour in Nijmegen, Netherlands (Oostenveld et al., 2011), and custom-written scripts in MATLAB (www.mathworks.com).

### Unsupervised MMN and P300 peak extraction

**Figure 1.**
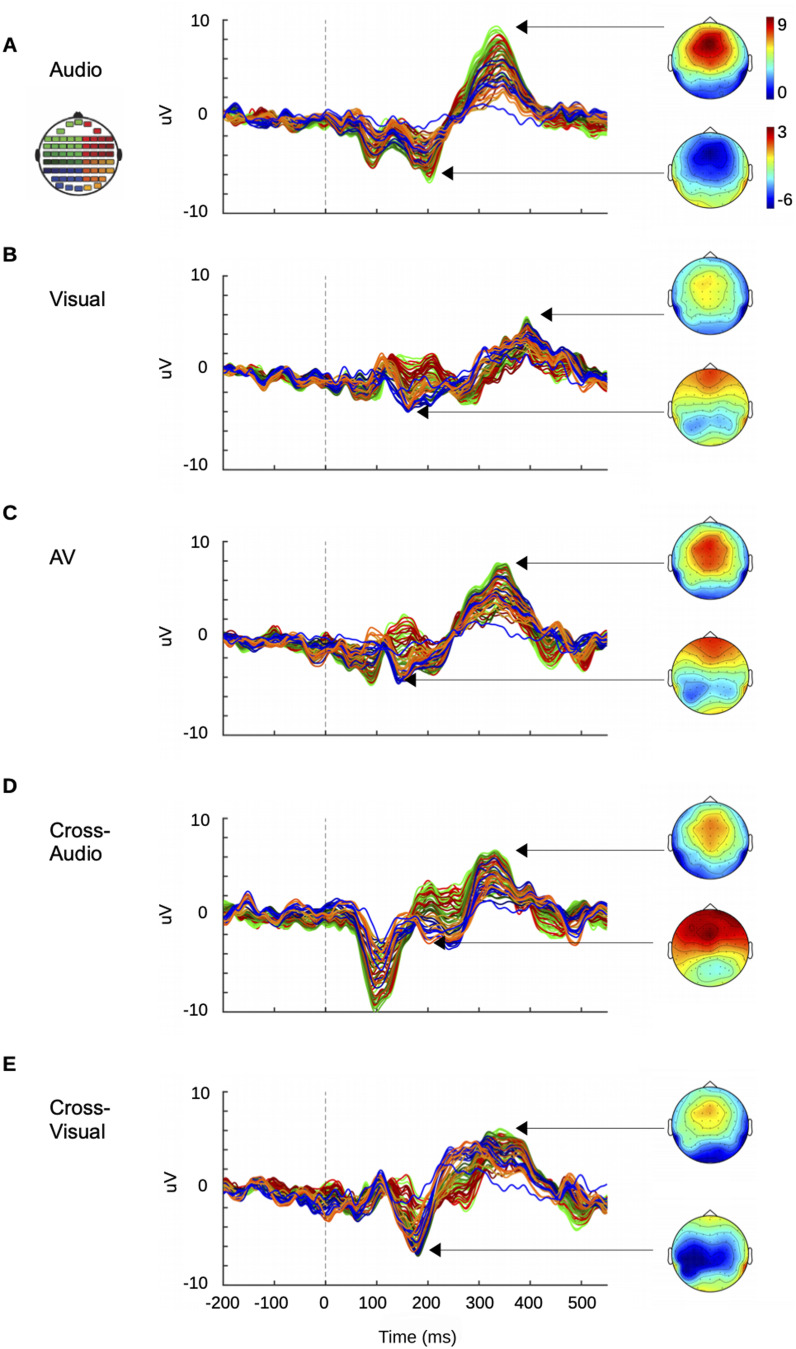
Grand average of difference ERPs (deviant-standard) plotted across 21 subjects for conditions (***A***) audio-only, (***B***) visual-only, (***C***) audiovisual, (***D***) cross-audio, and (***E***) cross-visual conditions. Stimulus onset is at 0 ms and the activity before that [−200 to 0 ms] corresponds to the prestimulus baseline. The topoplots indicate the distribution of the respective MMN and P300 peaks in the sensor space. The topoplot on the top-left displays the color code assigned to respective scalp channel locations used for plotting the ERPs in ***A–E***. For a curious reader, ERP from each condition, deviant, and standard are plotted at midline sensors in Extended Data Figure 1-1, along with SNR calculations in Extended Data Table 1-2.

MMN and P300 ERPs were observed in all five conditions: audio only (A), visual only (V), audiovisual (AV), cross-audio (CrA), and cross-visual (CrV) as seen in Figure 1, obtained by subtracting the grand average of standard waveforms from their respective deviant waveforms (waveforms of standard and deviant conditions in midline sensors are presented as Extended Data Figure 1-1). As evident from the peaks in the plots, precise estimation of MMN and P300 peak latencies/amplitudes when considering the activities of all sensors can be a challenging task as peaks from all sensors make the overall peak diffused. Averaging the EEG activity across sensors or selecting only specific central sensors for characterizing MMN and P300 peaks warrant only approximate estimations of their amplitudes and latencies. To eliminate all such researchers’ degrees of freedom, we identify the peaks in our study using a novel unsupervised data-driven method. Unlike the averaging method where a few peaks get lost in the process of averaging due to a set of sensors forming dipoles, our method effectively captures maximum variance in the waveforms across the entire sensor space. Due to the multivariate nature of this method, it is more reliable than the peak detection algorithms that essentially capture the local extrema for a single sensor time series (Dinh et al., 2021). For our analyses, we first pooled in trials (550 ms post-stimulus onset) from all the five conditions, i.e., A, V, AV, CrA, and CrV of the standard and oddball categories, and generated a common spatial filter using principal component analysis (PCA) on the difference (oddball-standard) waveforms. PCA computes the eigenvectors of the covariance matrix (“principal axes”) and sorts them by their eigenvalues (amount of explained variance). The loadings or “coefficients” obtained as the output of the MATLAB function *pca* were extracted to create the common spatial filter. To capture maximum variance in the data, we used the first four principal components as they together explained >80% of the data. In the ERP data (before dimensionality reduction), the signal-to-noise ratio expressed as 100*[(peak to peak amp-std dev)/peak to peak amplitude] was found to be ∼74–79% across all participants and conditions (presented as Extended Data Table 1-2). Thus, choosing a variance threshold of 80% retains all signal components that are relevant and ignores mostly the noise components.

10.1523/ENEURO.0251-23.2024.f1-1 Figure 1-1 Group averaged ERP plots of all conditions across midline channels – FPz, Fz, FCz, Cz, CPz, Pz, POz, Oz. Red-yellow hue represents averaged oddball trials along anterior to posterior axis and purple-cyan hue displays standard trials correspondingly. Download Figure 1-1, TIF file.

10.1523/ENEURO.0251-23.2024.t1-2 Table 1-2 The following table lists the signal-to-noise ratio (%) for each participant across all conditions. Please note the SNR was first computed for every channel and then averaged for each participant. Download Table 1-2, DOCX file.

**Figure 2.**
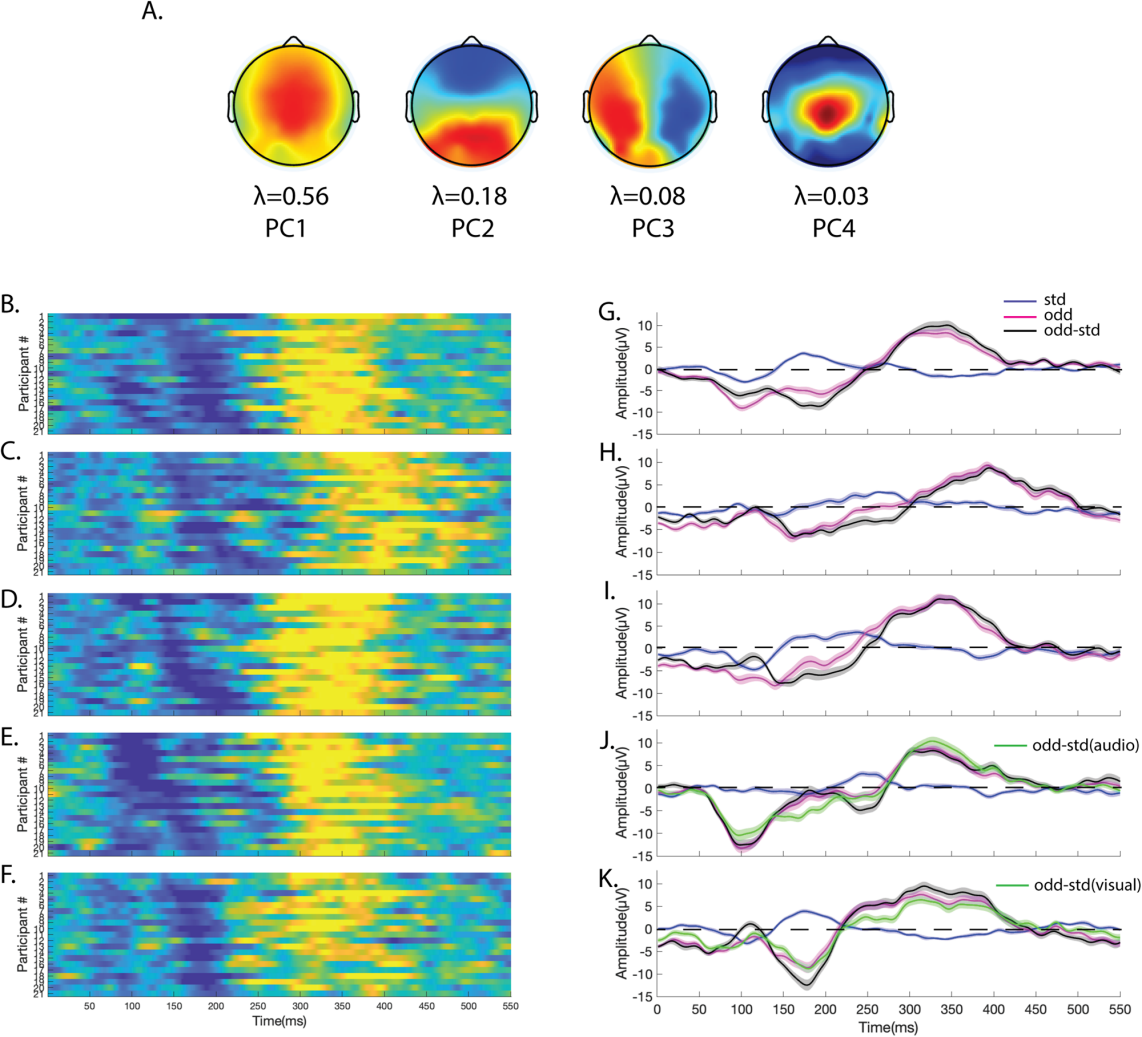
***A***, The topoplots correspond to the first four principal components (left to right) of the common spatial filter (Extended Data Figure 2-1) computed through PCA on the difference waveform of deviant minus standard trials together from all conditions. The *λ* values represent the fraction of variance explained by each principal component. The heatmaps on the left depict the time course of difference waveforms of individual participants after projection of the common spatial filter on the standard and deviant data of (***B***) audio-only, (***C***) visual-only, (***D***) audiovisual, (***E***) cross-audio, and (***F***) cross-visual conditions. On the right are the grand-averaged plots of the standard (blue), deviant (magenta), and difference (black) waveforms along with their shaded SEMs for (***G***) audio-only, (***H***) visual-only, (***I***) audiovisual, (***J***) cross-audio, and (***K***) cross-visual conditions.

Thus, choosing a variance threshold of 80% takes care of all signals that are relevant and ignores mostly the noise components. The common spatial filter obtained using PCA was then projected to the individual standard and oddball waveforms of each condition. The dimensionality of the data was hence reduced from timepoints × sensors × trials to timepoints × trials. A common spatial filter was used to optimize the differences between standard and deviant categories across all conditions. Figure 2*A* illustrates the “scores” of the first four principal components as topoplots along with their corresponding eigenvalues (*λ*) in the principal component subspace. After projecting the common spatial filter (first four PCs) on the standard and deviant waveforms of each condition, we computed the average of the four projected activities and subject-wise, inspected the presence of MMN and P300 ERPs in audio (Fig. 2*B*), visual (Fig. 2*C*), audiovisual (Fig. 2*D*), cross-audio (Fig. 2*E*), and cross-visual conditions (Fig. 2*F*). The difference wave (oddball − standard) for each condition projected on common spatial filter (Extended Data Figure 2-1*A*) didn't show much difference from projections on condition-specific filters (Extended Data Figure 2-1*B*) computed using PCA.

10.1523/ENEURO.0251-23.2024.f2-1 Figure 2-1 **A)** Common-spatial filter projected on the difference of oddball-standard data (averaged across all participants) for different conditions. **B)** Condition-specific filter projected on the difference of oddball-standard data (averaged across all participants) for different conditions. Download Figure 2-1, TIF file.

### Source localization

Source localization was performed to obtain the MMN and P300 generators in the brain across various modalities. The EEG data across all sensors were re-referenced with an average reference prior to source localization. Nineteen participants’ structural MRI data were resliced and segmented to identify the brain, skull, and scalp tissues. Two subjects’ MRI scans could not be obtained because of their incompatibility with the fMRI scanner. The origin (0,0,0) of all the T1 images was set to the anterior commissure. Participant-specific headmodel was computed using the OpenMEEG toolbox (Gramfort et al., 2010), using realistic conductivity values. The Polhemus data was imported to place the sensor locations on the head model of each participant. To obtain high accuracy of electrode positions, individual coregistration was employed by firstly visually marking the fiducials (nasion, left preauricular, and right preauricular) in the MRI volume and finally matching the marked points with the fiducial locations as per the Polhemus data. Next, the sources were placed in the segmented part of the head model at a distance of 5 mm from each other and the leadfield matrix was computed i.e., a transfer function between each source point and each electrode. Source localization of each individual was performed using their respective headmodel, leadfield, and electrode positions. For the inverse solution, covariance across all EEG sensors was computed for each condition. eLORETA, belonging to a class of current density measures to calculate the distribution of neuronal sources, was used to solve the inverse problem (Pascual-Marqui, 2007). eLORETA also generates the least amount of false-positives; hence, it is beneficial for exploratory source analysis, for example, where prior hypotheses regarding approximate locations may not be available (Halder et al., 2019). Lambda of 0.1 was used as the regularization parameter for the localization of P300 and MMN ERPs. After localization, each individual's source intensities were interpolated to their respective MRI volume. Further, to calculate the grand average of the source values, the interpolated images were normalized to a common structural template. Finally, we subtracted the voxel intensities of oddball and standard categories and the voxels having intensities >99th percentile were thresholded. This was done separately for each condition and hemisphere.

### Code and data accessibility

Codes used to analyze the data and generate the figures can be obtained from the bitbucket repository using the following link: https://bitbucket.org/cbdl/pe_erpandsourceanalysis/src/master/. Pre-processed and deidentifiable EEG data can be downloaded from the OSF repository: https://osf.io/v9nu5/. Copyright of the data and codes are held by the National Brain Research Centre, an autonomous institution of the Government of India.

## Results

### Temporal characterization of prediction error markers across modalities

**Figure 3.**
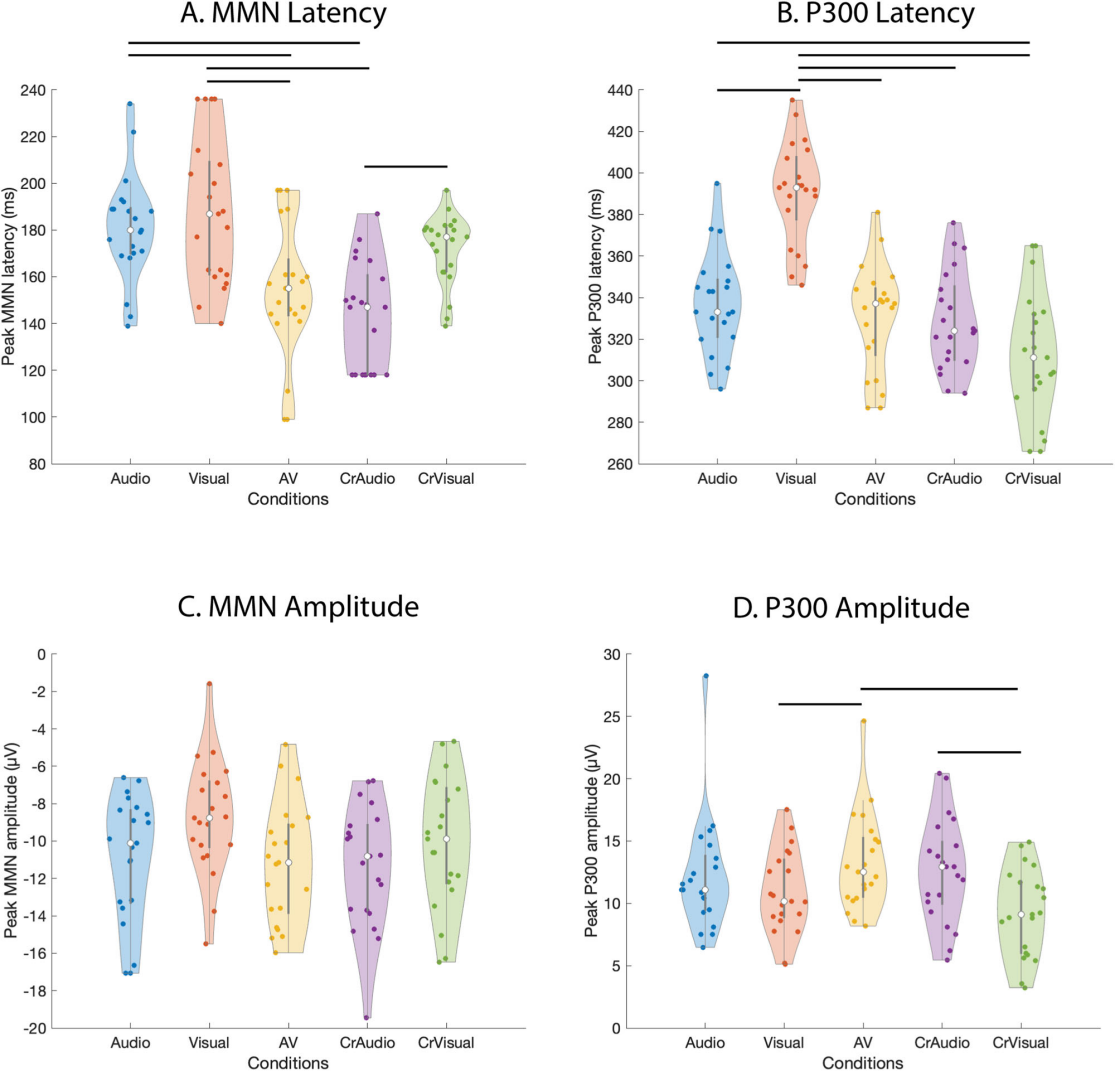
The violin plots represent the (***A***) MMN peak latencies corresponding to the (***C***) MMN peak amplitudes (maximum magnitude of the difference waveforms on the negative *y*-axis) of each participant. Similarly, the (***B***) P300 peak latencies corresponding to the (***D***) P300s peak amplitudes are plotted for each participant. The colored dots represent each participant's peak amplitude and peak latency values in µV and ms, respectively. The white dot at the center of the gray box in each violin represents the median of the data, and the gray box itself represents the inter-quartile range. The horizontal lines represent a significant difference (*p* < 0.05) between any two conditions obtained through Bonferroni-corrected post hoc analysis.

The P300 peaks were recognized in the difference waveforms as the most positive deflections and the MMN peaks as the most negative deflections (except for Cross-Audio (CrA)) where it was second most negative) across all modalities/conditions. We performed Student's *t* test on the dimensionality-reduced subject-level data to identify the temporal windows where MMN and P300 peaks in the deviant waveform showed significant differences in activities from their corresponding standard waveform in each condition. A timepoint-wise *t* test was conducted to identify the significant segments where, based on our data, significant activity (*p* < 0.001) corresponding to consecutive timepoints ≥10 ms were deemed significant. To maintain uniformity in the variances across conditions, the window length was restricted to 100 ms, centered at the peak. This window length was sufficient to include the descending and ascending limbs of MMN peaks and the ascending and descending limbs of the P300 peaks. The significant windows for MMN were found between 136 and 235 ms for audio only (Fig. 2*G*), 137–236 ms for visual only (Fig. 2*H*), 98–197 ms for audiovisual (Fig. 2*I*), 118–217 ms for cross-audio (Fig. 2*J*), and 121–220 ms for cross-visual (Fig. 2*K*) conditions. Similarly, the significant windows for P300 were found between 296 and 395 ms for audio-only (Fig. 2*G*), 346–445 ms for visual-only (Fig. 2*H*), 287–386 ms for audiovisual (Fig. 2*I*), 278–377 ms for cross-audio (Fig. 2*J*), and 264–363 ms for cross-visual conditions (Fig. 2*K*). For amplitude and latency characterization of MMN and P300, the minimum and maximum amplitudes, respectively, from the difference (deviant-standard) waveforms along with their corresponding latencies were extracted from the 100 ms windows for each subject across the audio only (A), visual only (V), audiovisual (AV), cross-audio (CrA), and cross-visual (CrV) conditions. Subsequently, the group mean and standard deviation of latencies and amplitudes were obtained for all the five conditions as listed in Table 2 for MMN and Table 3 for P300. We statistically investigated if the change of modality/condition indeed had an effect on the latencies and amplitudes of MMN/P300 peaks by conducting a repeated-measures analysis of variances (rmANOVA) on the subject-level data. The results revealed a significant effect of modality on the latencies of both MMN (*F*_(4,80)_ = 15.487, *p* < 0.0001, *η*^2^ = 0.436) and P300 (*F*_(4,80)_ = 33.390, *p* < 0.0001, *η*^2^ = 0.625), as well as on the amplitudes of MMN (*F*_(4,80)_ = 3.231, *p* = 0.016, *η*^2^ = 0.1391) and P300 (*F*_(4,80)_ = 6.8, *p* = 0.0001, *η*^2^ = 0.254). rmANOVA in this case is the best statistical test to check the interaction of modality with latencies/amplitudes because a simple ANOVA would otherwise inflate Type I error (false-positives) in between-subject effects and Type II error (false-negatives) in within-subject effects. An important point to keep in mind while using univariate rmANOVA is that the variances of the differences between all combinations of measurements should be equal. This is known as the sphericity (or circular) assumption, which is strongly assumed for within-subject rmANOVA statistics. If the sphericity assumption is violated, within-subject rmANOVA statistics are meaningless. We used Mauchly's test to verify that the assumption of sphericity was not violated (*p* > 0.05) in our comparisons. Furthermore, to employ a multiple comparisons correction that provides a good statistical power, post hoc analyses were performed using Bonferroni’s test to compare the peak MMN and P300 latencies/amplitudes between different conditions. As seen in Figure 3, the peak latency of audiovisual MMN (avMMN) was significantly faster than both audio MMN (*p* = 0.012) and visual MMN (*p* < 0.0001). Similarly, the peak latency of cross-audio MMN (craMMN) was significantly faster than both audio MMN (*p* = 0.0004) and visual MMN (*p* < 0.0001). Interestingly, cross-audio MMN was also significantly faster than cross-visual MMN (*p* = 0.001). Again for P300, the latencies of all non unisensory modalities—avP300, craP300, and crvP300—were significantly faster than the latency of the unisensory visual modality (*p* < 0.0001). The peak latency of crvP300 was also significantly faster than the peak latency of aP300 (*p* = 0.01). Between the unisensory modalities, the peak latency of vP300 was significantly slower than the peak latency of aP300 (*p* < 0.0001). The peak P300 amplitude of audiovisual condition was significantly higher than the peak P300 amplitudes of visual (*p* = 0.01) and cross-visual (*p* < 0.0001) conditions. Additionally, the peak P300 amplitude of cross-audio condition was significantly higher than the peak P300 amplitudes of cross-visual condition (*p* = 0.03). No significant differences were seen between the peak MMN amplitudes across all conditions.

**Table 2.** Table shows the mean ± SD of the peak MMN amplitudes and their corresponding latencies across all the subjects for audio-only, visual-only, audiovisual (AV), cross-audio (CrA), and cross-visual (CrV) conditions

MMN	Latency (ms)	Amplitude (μV)
Audio	180.81 ± 22.72	−10.91 ± 3.33
Visual	187.76 ± 31.38	−8.74 ± 3.06
AV	154.28 ± 28.88	−11.18 ± 3.19
CrA	142.90 ± 22.92	−11.37 ± 3.24
CrV	171.81 ± 15.19	−10.11 ± 3.49

**Table 3.** The table shows the mean ± SD of the peak P300 amplitudes and their corresponding latencies across all the subjects for audio-only, visual-only, audiovisual (AV), cross-audio (CrA), and cross-visual (CrV) conditions

P300	Latency (ms)	Amplitude (μV)
Audio	337.34 ± 24.45	12.13 ± 4.61
Visual	390.62 ± 24.53	10.87 ± 3.34
AV	330.38 ± 25.76	13.29 ± 3.87
CrA	328.81 ± 23.61	12.59 ± 4.12
CrV	312.24 ± 29.92	9.23 ± 3.50

### Spatial characterization of prediction error markers across modalities

**Figure 4.**
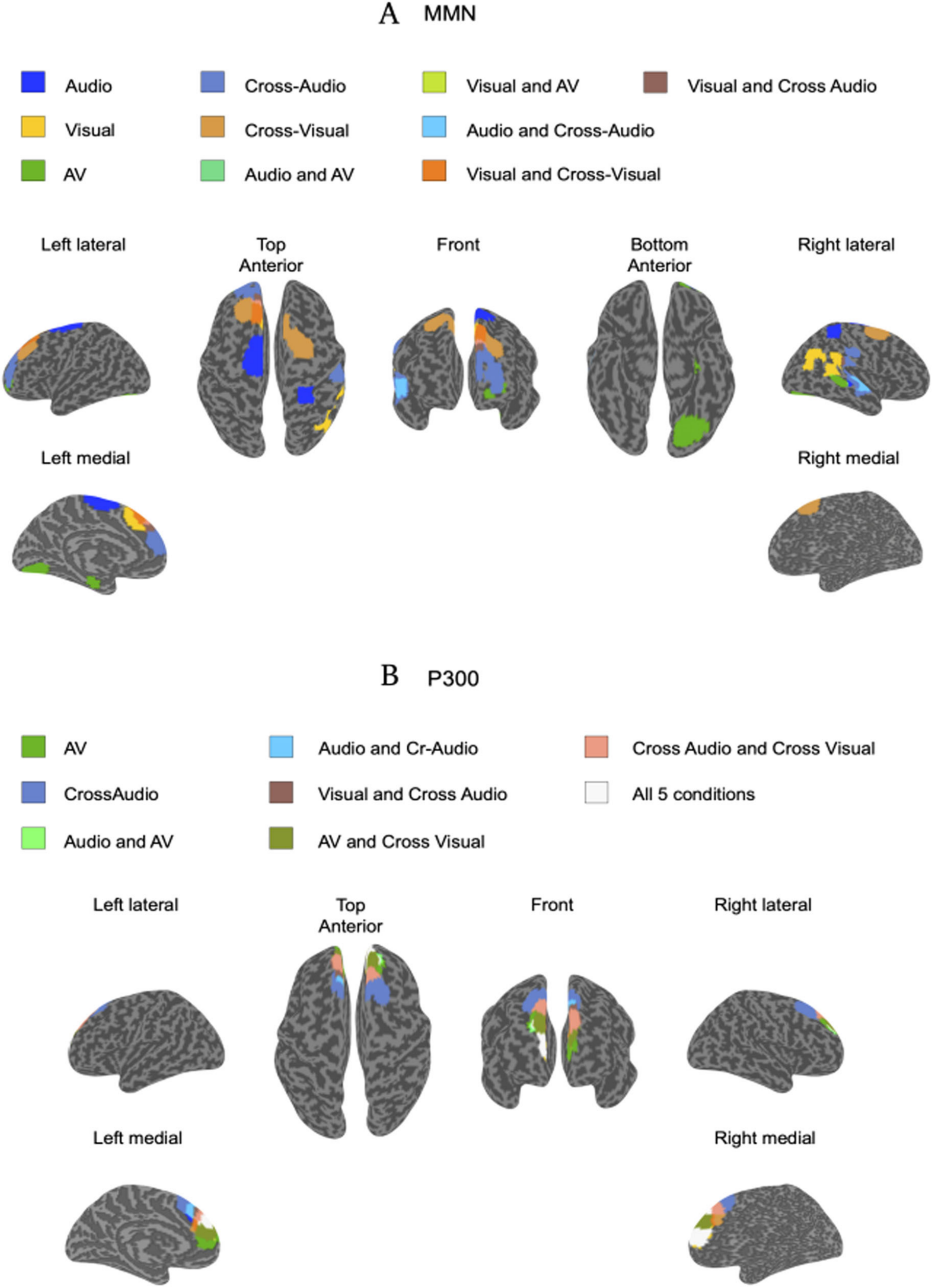
eLORETA source localization results using time-locked analysis (99th percentile and above) representing the underlying (***A***) MMN and (***B***) P300 sources for audio, visual, audiovisual, cross-audio, and cross-visual modalities.

The 100 ms windows of each modality were used to obtain the covariance matrices, separately for standard and oddball trials of MMN and P300. Using coregistration of individual participant MR data with their EEG sensor locations, we generated the source maps underlying MMN and P300 activity using eLORETA (details in Materials and Methods). The Brainnetome atlas was interpolated to the same common structural template as used for the normalization of the individual sources to locate the sources. As revealed in Figure 4, the MMN sources were distributed throughout the brain and were different for different modalities, while P300 displayed overlapping maps (locations in Table 4).

**Table 4.** The table lists the brain areas underlying the peak MMN and P300 activations across the audio-only, visual-only, and audiovisual conditions

Condition	MMN	P300
Audio-only	SFG, left superior frontal gyrus PrG, left precentral gyrus PrG, right precentral gyrus PCL, left paracentral lobule STG, right superior temporal gyrus MTG, right middle temporal gyrus aSTS, anterior superior temporal sulcus SPL, right superior parietal lobule	SFG, right superior frontal gyrus SFG, left superior frontal gyrus MFG, right middle frontal gyrus
Visual only	SFG, left superior frontal gyrus pSTS, right posterior superior temporal sulcus IPL, right inferior parietal lobule	SFG, right superior frontal gyrus SFG, left superior frontal gyrus
Audiovisual	MFG, left middle frontal gyrus MTG, right middle temporal gyrus Left fusiform gyrus Left parahippocampal gyrus pSTS, right posterior superior temporal sulcus IPL, right inferior parietal lobule MVOcC, left medioventral occipital cortex LOcC, left lateral occipital cortex	SFG, right superior frontal gyrus SFG, left superior frontal gyrus MFG, right middle frontal gyrus MFG, left middle frontal gyrus Left cingulate gyrus
Cross-audio	SFG, left superior frontal gyrus MFG, left middle frontal gyrus STG, right superior temporal gyrus MTG, right middle temporal gyrus IPL, right inferior parietal lobule	SFG, right superior frontal gyrus SFG, left superior frontal gyrus MFG, right middle frontal gyrus
Cross-visual	SFG, right superior frontal gyrus SFG, left superior frontal gyrus MFG, left middle frontal gyrus MFG, right middle frontal gyrus	SFG, right superior frontal gyrus SFG, left superior frontal gyrus

The left superior frontal, paracentral, right superior, and middle temporal gyri, bilateral precentral gyrus, and bilateral superior parietal lobule were found to underlie the MMN response in the audio-only condition; the left superior frontal gyrus and right posterior superior temporal sulcus and inferior parietal lobule elicited the MMN in the visual-only condition; and the audiovisual condition yielded sources that were located in the left middle frontal gyrus, fusiform area, parahippocampal gyrus, medioventral and lateral occipital cortex, and right middle temporal gyrus, posterior superior temporal gyrus, and inferior parietal lobule. In the cross-modal conditions, left superior and middle frontal gyri, right superior and middle temporal gyri, and inferior parietal lobule were found in cross-modal audio condition, while the cross-modal visual condition was elicited from the bilateral middle and superior frontal gyri (details in Table 4). In summary, all conditions had distributed source maps, however, some commonalities were present, such as all conditions were elicited from the frontal regions and all conditions except cross-visual included the parietal regions and importantly, different clusters in the temporal area.

In contrast, source analysis of P300 revealed overlapping frontal areas for all the five conditions (as illustrated in Fig. 4*B*). P300 was elicited at the bilateral frontal gyrus in all five conditions, the right middle frontal gyrus in audio-only and cross-audio task, and the bilateral middle frontal and cingulate cortex in the AV (details in Table 4).

## Discussion

We employed two-stimulus oddball tasks using audio-alone (A), visual-alone (V), synchronous audiovisual (AV), cross-modal audio (CrA), and cross-modal visual (CrV) conditions to evoke MMN and P300 components—ERP-level markers of prediction error processing (Chennu et al., 2013; Stefanics et al., 2014; Calcus et al., 2015; Aitchison and Lengyel, 2017; Banellis et al., 2020). Addressing our first hypothesis, we elucidate how the presence of multimodal and cross-modal audiovisual contexts speed up the processing speeds of prediction errors at the middle (MMN) and later (P300) stages of mismatch information processing. Our study provides a novel data-driven method to accurately confirm the understanding of cortical information processing and, hence, can serve as a powerful tool to formulate “internal models” trying to understand prediction error processing at different stages/hierarchies in the brain (Wacongne et al., 2012). In the statistical learning literature, PCA is recognized as an unsupervised approach that involves the diagonalization of the covariance matrix computed from two random variables without explicit assumption of a specific relationship (model) between them, for example, in the case of regression (which assumes a linear model; Jollife, 2002). No single study before ours has demonstrated this in healthy humans so explicitly, involving both the prediction error markers across different modality conditions and pinpointing the exact temporal scale when modality-specific processing is completed in the cortex. While addressing our second hypothesis, we found that the modality-specific and modality-independent nature of MMN and P300 is complex. As we move from MMN to P300, we see an overall loss in the modality specificity in terms of cortical sources. While MMN sources had a significant overlap over frontal cortical locations, unlike P300 sources, there were also areas such as in temporal and parietal regions specific to a particular modality. An important caveat to note is that while PCA allows the unsupervised comparison, it is also possible if the threshold for variance is not empirically based, noise in one condition leaks into the common spatial filter, thus creating an unnecessary bias for latency computation. So, it is advisable that the threshold eigenvalues for signal rank determination are empirically motivated.

### Multisensory facilitation of MMN and P300

Previous studies have evidenced auditory cortical activity to operate at a faster timescale than visual cortical activity (Williams et al., 2004; Kumar et al., 2020). As expected, in our study, both auditory mismatch negativity (aMMN) and aP300 ERPs were faster than visual MMN (vMMN) and vP300 ERPs, respectively. But what happens when these two modalities come together in different combinations still needs to be deciphered. When audio and visual oddballs are presented synchronously against a background of synchronous audiovisual standards, avMMN is significantly faster than both aMMN and vMMN. As the mismatch signal further propagates onto the P300 level, aP300 and avP300 attain comparable latencies, avP300 still being significantly faster than vP300. This suggests the presence of multisensory facilitation where the faster auditory component in the AV oddball temporally facilitated its slower visual component, which speeded up the process of change detection in the audiovisual condition. On the other hand, despite having the same audio deviant in cross-audio condition as used in the audio-only condition (and the same visual deviant in cross-visual condition as presented in the visual-only condition), the latency of craMMN was significantly shorter than aMMN while that of crvP300 was significantly shorter than vP300, indicating that the processing speed of oddballs is not only determined by the modality of the oddball itself but by that of the background standard as well.

The speeded appearance of the prediction error markers in the audiovisual condition can possibly be attributed to an early coupling in the information processing hierarchy between the constituent unisensory audio and unisensory visual deviant stimuli during an early sensory processing stage (e.g., at N100; Van Wassenhove et al., 2005; Stekelenburg and Vroomen, 2007). An alternate account could be that the unisensory systems operate largely independently to extract feature-level information, before multisensory convergence and integration marked by avMMN at the superior temporal gyrus and middle temporal gyrus, the higher-order association areas (Callan et al., 2004; Beauchamp, 2005). If there were no intersensory coupling at the sensory-perceptual level, the MMN for combined visual and auditory deviants should reflect a linear summation of the individual auditory and visual MMNs (Molholm et al., 2002, for auditory and somatosensory deviants). In our results, however, we saw a clear nonlinearity in the multisensory response where the amplitudes of the avMMN and avP300 were smaller than the sum of amplitudes of aMMN and vMMN as well as that of aP300 and vP300, respectively (Fig. 3*C,D*). This suggests that the aMMN and aP300 responses were minimized when paired with the concurrent visual stimulation, which can be attributed to the bimodal nature of our audiovisual stimuli as the presence of two stimuli gives the brain more flexibility to choose between deviant features to process the AV oddballs efficiently and, hence, conserve its neural resources during change detection in accordance to the free energy principle (Friston, 2010). To minimize refractoriness in the sensory cortical feature-specific neurons (Jacobsen and Schröger, 2001), there may also be a switch between which features the brain attends to, during the course of the oddball experiment. In the future, comparing audio and visual deviant stimuli against a matched audiovisual standard can further explain if the deviant stimuli alone can bring forth any change in the processing speed of prediction errors and also mitigate differences arising due to different baseline noise in standards. To better understand the nature and extent of audiovisual interactions, it will be essential to design advanced paradigms that form meaningful stimuli in both unisensory and multisensory constructs.

### Loss of specificity from MMN to P300

Source localization results for MMN showed heterogeneous activations in the brain across different sensory modalities (as shown in Fig. 4*A*). From these results, we concur that mostly the primary and secondary sensory areas distributed throughout the brain along with other higher cortical areas are employed during the process of early prediction mismatch, based on the modality. In traditional literature, MMN is widely considered a “perceptual” prediction error signal carrying important novel information that initiates a call for further processing of prediction mismatches (Mäntysalo and Näätänen, 1987; Schröger, 1997; Escera et al., 2000). Such novel information should necessarily feed into the higher cognitive processing areas for further evaluation, as is reflected by the occurrence of the late event-related potential, P300 (Bledowski et al., 2004b; David and Linden, 2005). Our source results revealed common source activations for P300 localized around the fronto-central regions for audio-only, visual-only, audiovisual, cross-audio, and cross-visual conditions (as shown in Fig. 4*B*). Common brain generators for P300 across modalities suggest that this neural marker might particularly be responsible for domain-general higher evaluative processes like keeping a count of the number of oddballs as in our paradigm, after a change detection signal has been relayed by MMN generators of corresponding modalities in the brain. A caveat to note here is that the MMN stage does not necessarily reflect complete modality dependence, and some amount of overlapping cortical sources is probably responsible for its generation. Nonetheless, our results highlight that the existence of modality-dependent cortical modules almost disappears only at P300 stage suggesting a reorganization of brain regions along the temporal hierarchy of prediction error processing (MMN followed by P300) as the mismatch information flows up the spatial hierarchy of the brain (sensory to higher-order regions). Though such a transition may appear unsurprising in terms of neurocognitive processing, we questioned at what stage in the information processing hierarchy is the modality specificity to the sensory stream lost? We found that the processing of prediction error becomes completely amodal at the P300 level itself and is not something that is attained at further later stages during the reorienting negativity (RON) that is elicited at ∼400–600 ms (Correa-Jaraba et al., 2016; Ungan et al., 2019).

An important aspect of our present study is that the hierarchical stages of prediction error processing can be explained by multiple underlying internal models of error propagation (Aitchison and Lengyel, 2017). A detailed neuronal model of the auditory, visual, and audiovisual cortices, based on the underlying processes of predictive mismatch that account for the critical features of MMN, P300, and RON could better explain the process of temporal facilitation and supra-additivity in the audiovisual modality (Molholm et al., 2002; Pattamadilok and Sato, 2022). Our empirical investigations in the context of hierarchical processing of prediction errors would have implications beyond the theoretical domain as well. Based on the models of MMN and P300 responses from patients with disorders of consciousness like in vegetative and minimally conscious states (Daltrozzo et al., 2007; Boly et al., 2011), attention (Polich, 2007; Szuromi et al., 2011), and schizophrenia (Blackwood, 2000; Erickson et al., 2016), researchers can isolate the deficits in predictive information flow that might underlie these states of profound cognitive and neurological dysfunction. Such foundational advances can be of extreme value to clinical neuroscience researchers. From a neural networks’ perspective, two well-known attentional networks, the dorsal attention network (DAN) and the ventral attention network (VAN), have been largely reported in oddball studies (Clark et al., 2000; Stevens et al., 2000; Bledowski et al., 2004a). The VAN in particular has been exclusively involved in the detection of deviant stimuli (Palaniyappan and Liddle, 2012; Kim, 2014) and is activated at both MMN and P300 stages (Justen and Herbert, 2018). Further connectivity analysis between the regions underlying the VAN during MMN and P300 latencies across various modalities can further our understanding of the function of this important attentional network beyond recent evidence in the frequency domain (Ghosh et al., 2021). Although a few studies have also attempted to draw a relationship between prestimulus brain states and P300 (Reinhart et al., 2011; Karch et al., 2016), a detailed source connectivity analysis will be informative about the mechanisms of P300 and MMN generation, thus providing an important direction to future research.

## Synthesis

Reviewing Editor: Ifat Levy, Yale School of Medicine Decisions are customarily a result of the Reviewing Editor and the peer reviewers coming together and discussing their recommendations until a consensus is reached. When revisions are invited, a fact-based synthesis statement explaining their decision and outlining what is needed to prepare a revision will be listed below. The following reviewer(s) agreed to reveal their identity: NONE.

In their rebuttal and revision, the authors have addressed most of my previous concerns, and I would like to thank them for their additional explanations.

However, their reply to issue (1) remains partly unconvincing. The additional information provided is very helpful, but it actually highlights my original point:

I assume that a substantial part of the 80% variance explained across conditions reflects differences between conditions. Reducing shared and non-shared variance from the different condtions to a set of common factors necessarily means that conditions differ in terms of their factor loadings. These differences *may* reflect functionally interpretable effects, but this is by no means guaranteed.

Hence my recommendation remains that original (i.e. non-difference) ERPs from the different conditions should be presented in order for the reader to get an impression of when and where differences appear in the original sensor space. The current Figure 1 is only moderately helpful in this respect, as it presents difference ERPs across all channels at a very reduced scale. Given the exceptionally large body of literature on both MMN and P300 components, I do not think that selecting channels for this primarily descriptive purpose is overly problematic.

## Author Response

1 Response to reviews We thank the anonymous reviewers for insightful comments that have strengthened the quality of the manuscript. Here we address each of the comments in the order they were raised.

Synthesis of Reviews:

Computational Neuroscience Model Code Accessibility Comments for Author (Required): please include a statement in the Materials and Methods section, under the heading "Code Accessibility", indicating whether and how the code can be accessed, including any accession numbers or restrictions, as well as the type of computer and operating system used to run the code. "2.8 Code and data accessibility:" section is added.

Synthesis Statement for Author (Required):

This manuscript reports results from a small-scale EEG study. It describes a combined analysis of the mismatch negativity (MMN) and the P300 components of auditory-visual ERPs in healthy human volunteers. Using a principal component analysis (PCA) approach combined with a distributed inverse solution, the authors report confirmatory evidence for the notion that the two components reflect mechanisms of prediction-error detection at different hierarchical levels of sensory processing.

The manuscript is well written, and the simultaneous assessment of MMN and P300 components within the same analytic framework makes this a potentially interesting contribution to the literature. However, reviewers had significant concerns that must be addressed, as detailed below, before it can be determined to what extent the proposed method advances the field.

2 Thank you for giving us the opportunity to address comments and resolve the concerns.

1. It seems that substantial differences between the experimental conditions are essentially disregarded during the PCA step, only to be analyzed and interpreted in detail in subsequent analyses.

This may be particularly problematic given that data from all conditions are used to generate a common spatial filter capturing "more than 80%" of variance (p.10), with this filter then being applied separately to standard and oddball ERPs from the individual conditions. As an example for the potential problems of this approach, consider the cross-modal conditions which likely require more complex and more distributed computations than the unimodal conditions, including but not limited to omitted-stimulus responses in the standard modality. In this context, it appears trivial to observe latency and amplitude differences between conditions which have previously been reduced to less than a handful of factors. At the very least, the authors should provide a description of the ERPs from the different conditions so that readers may appreciate whether and how commonalities and differences are captured by the proposed method.

We agree that the cross-modal conditions likely require more complex and more distributed computations than the unimodal conditions because of reasons that are also evident from our ERP plots (Figure 1) where, for instance, the cross-audio condition doesn't have a clear MMN peak. The ERP plot shows a large N100 peak followed by a more prominent N200 peak with a dispersed peak between the two, which most likely is the MMN peak. Characterizing the amplitude and latency of such peaks is challenging because a) taking an average across all channels would further disperse the peak and b) selecting channels that effectively contribute to the peak in an unbiased way is a daunting task. Therefore, we resort to an unsupervised method to solve this problem. For our unsupervised approach, we have 3 used a common spatial filter for PCA to optimize for the MMN/P300 differences across conditions. As suggested by the reviewer, using different filters for different conditions does guarantee finding differences in the peak characteristics between the conditions by maximizing the signal variance but that may not necessarily be related to the difference between the responses due to the stimulus condition itself. Hence, we use a common spatial filter that explains more than 80% of the variance in the signal which is almost the same as the % of signal components in our ERP data (before PCA) which we validated by computing the signal-to-noise ratio for all our participants in each condition which can be found in the table S1 of Supplementary material We have further explained the use of our unsupervised approach in our response to comment 3. As seen in the ERP plots of Figure 1, MMN was found as the trough after 100 ms indicating the contribution of different sensors across different conditions as illustrated in the topoplots. However, P300 in all conditions had similar fronto-central topographies.

2. The description of both the methods and the results of the source localization analyses remain rudimentary. It seems likely that sources were localized on the basis of the full difference ERPs and not the PCA-filtered data, but reviewers were not able to find this information in the manuscript. Moreover, given the predominance of sources identified on the medial surfaces of both hemispheres, a separate panel of these surfaces should be included in Fig. 4. In their current form, it is not clear how the present data support the claim of supramodal prediction-error responses emerging over time.

It is true that sources were localized based on full difference ERPs and not the PCA filtered data because PCA filtered data does not retain the original sensor space information. Hence, source localization with data that has reduced dimensionality in the sensor space itself is not appropriate.

4 The purpose of the PCA analysis was to counter the problem of comparing different channels in different conditions in the first place with an unsupervised approach (more on this in the following response).

To be more clear, the following is added to the the source localization methods section 2.7 - "The EEG data across all sensors were re-referenced with an average reference prior to source-localization." and "Source localization of each individual was performed using their respective headmodel, leadfield and electrode positions. For the inverse solution, covariance across all EEG sensors was computed for each condition. eLORETA, belonging to a class of current density measures to calculate the distribution of neuronal sources, was used to solve the inverse problem (Pascual-Marqui, 2007)." We now have added medial slices for P300 and MMN source visualizations clearly showing distributed MMN sources and overlap of P300 sources across all conditions in frontal region.

3. It is not clear whether the authors' approach is indeed 'unsupervised': a completely unsupervised selection of principal components (based on an arbitrary variance criterion) and maxima and minima in the filtered data would likely result in even larger problems in more complex datasets In the statistical learning literature PCA is recognized as an unsupervised approach since it involves diagonalization of the covariance matrix computed from two random variables without explicit assumption of specific relationship (model) between them e.g in case of regression (which assumes a linear model) and which is a supervised approach (Jollife, 2002).

Furthermore, variance criterion remains the same across all conditions and was chosen based on signal to noise estimate from the raw ERP data. SNR expressed as 100*[(peak to peak amp-std dev)/ peak to peak amplitude] was found to be around 74-79% across all participants and conditions. Thus choosing a variance threshold of 80% takes care of all signals that are 5 relevant and ignores mostly the noise components. In supplementary Information we have now added the SNR computations. (Table S1) Partici pant # Audio only Visual only AV Cross-audio Cross-visual 1 74.87 75.37 76.08 74.25 75.42 2 75.32 75.22 75.19 76.52 75.99 3 73.11 72.92 73.59 75.34 72.43 4 73.83 75.05 75.82 77.21 78.13 5 77.36 74.66 74.72 74.70 76.32 6 75.11 76.33 74.97 75.46 74.50 7 74.57 75.57 75.34 78.19 76.04 8 74.94 76.04 75.08 76.73 77.43 9 77.12 76.87 75.66 75.51 76.37 10 76.41 74.98 75.97 77.80 74.94 11 77.43 78.13 75.87 76.11 76.34 12 75.01 77.12 73.66 77.08 75.84 13 76.39 76.52 75.21 77.35 74.39 14 76.17 77.35 75.51 76.57 75.91 15 77.65 76.75 74.62 77.43 77.93 16 75.28 75.15 75.28 77.95 76.82 17 74.58 74.26 76.26 76.98 74.21 18 75.85 76.57 75.99 75.95 76.67 19 75.36 77.80 75.41 77.98 77.21 20 75.71 77.60 77.44 76.90 76.77 6 21 78.07 78.90 76.04 77.71 76.97 Jollife, I. T (2002) Principal Component Analysis.

We have added the following sentences in Discussion: "In the statistical learning literature, PCA is recognized as an unsupervised approach which involves the diagonalization of the covariance matrix computed from two random variables without explicit assumption of specific relationship (model) between them e.g., in case of regression (which assumes a linear model) (Jollife, 2002)." 4. It will be helpful if the authors could discuss further why the latency in the cross-audio condition is shorter compared to the latency in the cross-visual condition.

The peak MMN latency in the cross-audio condition is significantly shorter compared to the peak latency of cross-visual condition MMN.

We think there could be two possible reasons for this: a) As also discussed in the manuscript, the processing speed of auditory modality is faster than visual modality (Kumar et al., 2020; Williams et al., 2004) leading to the detection of auditory oddballs/ prediction errors faster than visual oddballs/prediction errors. b) The latency of MMN/P300 is dependent on the saliency/discrimination difficulty of oddballs (Magliero et al., 1984; Patel & Azzam, 2005; Polich, 2007). In the cross-audio condition, an audio oddball is presented against a standard of visual standards. Here, the only audio input is the audio oddball. In the cross-visual condition, a visual oddball is presented against a standard of audio standards. In this condition however (which may be an experimental limitation), along with the audio oddball is presented a cross fixation on the screen which does provide some minimum visual stimulation constantly. As the discrimination difficulty reduces in the latter case (presence of oddball could either mean 7 hearing a sound or seeing a '+' and the brain can conveniently choose to attend to just one option), the brain probably prioritizes the processing of cross-audio oddball because of greater associated novelty.

We have added the following sentence to elaborate on point (b): "On the other hand, despite having the same audio deviant in cross-audio condition as used in the audio only condition (and the same visual deviant in cross-visual condition as presented in the visual only condition), latency of craMMN was significantly shorter than aMMN while that of crvP300 was significantly shorter than vP300, indicating that the processing speed of oddballs is not only determined by the modality of the oddball itself but by that of the background standard as well."

Minor issues:

5. p.8, first paragraph: Participants' task was to count the number of deviant stimuli per block. The authors should discuss potential effects of this setting on the two components of interest, as the MMN is also seen for prediction errors that remain unconscious, whereas P300 is more closely related to conscious awareness of prediction errors. Also, performance on the counting task should be reported and/or exclusion criteria based on this variable should be specified.

We agree that MMN must be seen in both oddball counting and passive viewing tasks since MMN may not be related to conscious awareness whereas P300 is related to conscious awareness only. The question whether computing MMN in counting tasks like ours, where conscious awareness is a requirement, will have any potential effect on the ERP cannot directly be answered because we don't have data of MMN without the counting context.

However, the subtraction of standard trials from oddball trials while computing latencies should take care of the common factors that affect both MMN and P300.

8 We have now included a table with the performance of each participant in all five task conditions and added in the Supplementary Material (Table S2).

The participants in our task had to report the total number of oddballs at the end of each block (randomized across blocks) which was either deemed 'correct' or 'incorrect' based on whether or not the count of oddballs matched with the actual number of oddball trials present in that block. Since there were 4 blocks from each task condition, 100% accuracy means the participants correctly counted oddballs in all the 4 blocks, 75% accuracy means the participants correctly counted oddballs in 3 out of the 4 blocks and 50% accuracy means participants correctly counted oddballs in 2 out of the 4 blocks. No participant had % accuracy less than 50 in any condition.

Therefore, to preserve the number of trials from each participant across conditions, no blocks were excluded based on behavioral performance.

Please note that the performance details of the first participant are not available.

Performance (% accuracy) across task conditions Participant # Visual only Audio only Audio-visual Cross-audio Cross-visual No. of years of musical training 1 - - - - - - 2 100 100 100 100 75 2 years 3 100 100 75 75 100 None 4 100 100 75 50 100 None 5 100 100 100 100 100 10 years 6 100 100 100 75 100 None 7 75 100 100 100 100 None 8 100 100 100 100 100 None 9 100 100 75 75 100 None 10 100 50 100 50 100 None 11 75 100 100 75 100 7 years 12 100 100 75 75 100 None 13 75 75 100 100 100 None 9 14 100 100 75 100 100 None 15 75 100 100 100 100 None 16 50 100 50 75 75 6 years 17 100 75 100 100 100 None 18 100 100 100 100 100 None 19 100 100 100 100 100 7 years 20 75 100 100 100 100 None 21 100 100 100 75 100 None 22 75 75 50 100 100 None 6. p.9, Preprocessing: Why was the low-pass filter applied to epoched instead of continuous data? And what was the interval used for baseline correction? Also, the following sentence regarding how trial numbers were equated between standard and oddball conditions was confusing: on p.8, the authors state that there were 400 trials per each of five condition, 56 of which were oddballs - how does this fit with 885 deviant trials mentioned here? We corrected and repeated our analysis pipeline by applying the low pass filter before epoching. The interval used for baseline correction is [-500 0] where 0 is the onset of the stimulus. For each participant and condition separately, the number of standard trials were equalized to the number of oddball trials. The following edits have been made in the manuscript - "EEG was acquired from 22 participants out of which data from 1 participant was discarded due to noisy recordings. The raw data of the remaining 21 participants were imported and each block was epoched. A high pass filter of 0.1 Hz was applied to the data to remove slow drifts in the signal. The data were visually inspected further and 1 channel (F4 in 3 subjects) was interpolated to neighboring electrodes. To identify and remove blink artifacts from the data, independent component analysis (ICA) was employed for each block. Only the blink component obtained as the independent component (IC) from eye regions was visually identified and subsequently that IC was rejected. Furthermore, all blocks were subjected to a low pass 10 filter of <45 Hz and then, each block was epoched to trials of [-500 550] ms where 0 ms marked the onset of the stimulus. The trials were further divided into standard and oddball categories, baseline correction was applied using the pre-stimulus activity and the data was re-referenced to linked mastoids.

Trials with signal amplitude greater than 100µV and lesser than -100µV were removed and at most 6% of all oddball trials were discarded per subject.

Then we equalized the number of oddball and standard trials within each separate condition for each participant by randomly selecting standard trials.

Following are the total number of oddballs or standard in each condition with mean and standard deviation across participants - Audio only condition was 1416 (mean = 67.4286, S.D. = 5.835), Visual only was 1397 (mean = 66.5238, S.D. = 8.2257), AV was 1450 (mean = 69.0476, S.D. = 2.8544), CrossAudio was 1468 (mean = 69.9048, S.D. = 1.5781) and CrossVisual was 1461 (mean = 69.5714, S.D. = 1.1650). All analyses were done using the MATLAB based FieldTrip toolbox developed at Donders Institute for Brain, Cognition and Behaviour, in Nijmegen, Netherlands (Oostenveld et al., 2011) and custom-written scripts in MATLAB (www.mathworks.com)." The Results also underwent changes slightly without change in the overall pattern. All Figures (Fig 1-4) were redone with new preprocessing steps and Results sections where numbers were reported (Tables 2,3,4) are updated.

7. p.11, Source localization: Given that an average reference is used for source localization, is it (mathematically) necessary to use a local reference for the PCA step? Most of the classical MMN and P300 studies use mastoid and nose re-referencing (Näätänen et al., 2007) to identify the peaks in the waveforms and to avoid seeing dipoles resulting from average re-referencing. It is however, mathematically necessary to use average referencing for source localization because of a small model error at each channel in the forward model determining how the sources project to the channels. Average reference spreads the forward modeling error evenly across channels ensuring that no one channel is weighted more strongly during source estimation. It is also 11 well explained in the Fieldtrip toolbox FAQ, as we used the toolbox for source estimation.

8. p.17: New labels are introduced here for the different conditions; would be better to stick to the same labels throughout the manuscript.

The labels for AV, A, V, CrA and CrV are made consistent throughout the manuscript. Table 1 is updated with these labels.
