# Supplementary material for: Unsupervised Characterization of Prediction Error Markers in Unisensory and Multisensory Streams Reveal the Spatiotemporal Hierarchy of Cortical Information Processing
Source: eNeuro. 2024 May 2;11(5):ENEURO.0251-23.2024. doi: 10.1523/ENEURO.0251-23.2024 (PMC11069433; doi:10.1523/ENEURO.0251-23.2024)
Supplement: Table 1-1 — Counting accuracy of participants in different task conditions. Download Table 1-1, DOCX file. [file eneuro-11-ENEURO.0251-23.2024-s004.docx]

**Table 1-1.** Counting accuracy of participants in different task conditions

| **Performance (% accuracy) across task conditions** | | | | | | |
| --- | --- | --- | --- | --- | --- | --- |
| **Participant #** | ***Visual only*** | ***Audio only*** | ***Audio-visual*** | ***Cross-audio*** | ***Cross-visual*** | **No. of years of musical training** |
| 1 | - | - | - | - | - | - |
| 2 | 100 | 100 | 100 | 100 | 75 | 2 years |
| 3 | 100 | 100 | 75 | 75 | 100 | None |
| 4 | 100 | 100 | 75 | 50 | 100 | None |
| 5 | 100 | 100 | 100 | 100 | 100 | 10 years |
| 6 | 100 | 100 | 100 | 75 | 100 | None |
| 7 | 75 | 100 | 100 | 100 | 100 | None |
| 8 | 100 | 100 | 100 | 100 | 100 | None |
| 9 | 100 | 100 | 75 | 75 | 100 | None |
| 10 | 100 | 50 | 100 | 50 | 100 | None |
| 11 | 75 | 100 | 100 | 75 | 100 | 7 years |
| 12 | 100 | 100 | 75 | 75 | 100 | None |
| 13 | 75 | 75 | 100 | 100 | 100 | None |
| 14 | 100 | 100 | 75 | 100 | 100 | None |
| 15 | 75 | 100 | 100 | 100 | 100 | None |
| 16 | 50 | 100 | 50 | 75 | 75 | 6 years |
| 17 | 100 | 75 | 100 | 100 | 100 | None |
| 18 | 100 | 100 | 100 | 100 | 100 | None |
| 19 | 100 | 100 | 100 | 100 | 100 | 7 years |
| 20 | 75 | 100 | 100 | 100 | 100 | None |
| 21 | 100 | 100 | 100 | 75 | 100 | None |
| 22 | 75 | 75 | 50 | 100 | 100 | None |
