# Supplementary figures and images for: Unsupervised Characterization of Prediction Error Markers in Unisensory and Multisensory Streams Reveal the Spatiotemporal Hierarchy of Cortical Information Processing
Source: eNeuro. 2024 May 2;11(5):ENEURO.0251-23.2024. doi: 10.1523/ENEURO.0251-23.2024 (PMC11069433; doi:10.1523/ENEURO.0251-23.2024)

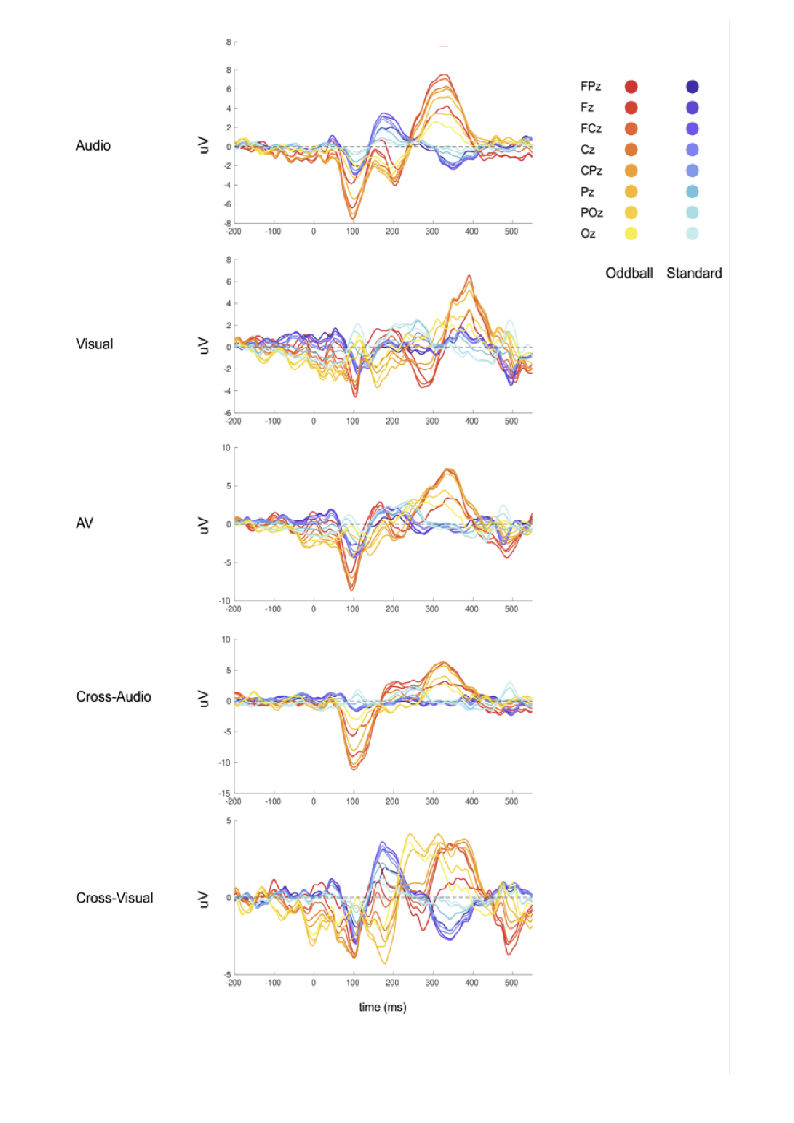

Supplement: Figure 1-1 — Group averaged ERP plots of all conditions across midline channels – FPz, Fz, FCz, Cz, CPz, Pz, POz, Oz. Red-yellow hue represents averaged oddball trials along anterior to posterior axis and purple-cyan hue displays standard trials correspondingly. Download Figure 1-1, TIF file. [file eneuro-11-ENEURO.0251-23.2024-s002.tif]

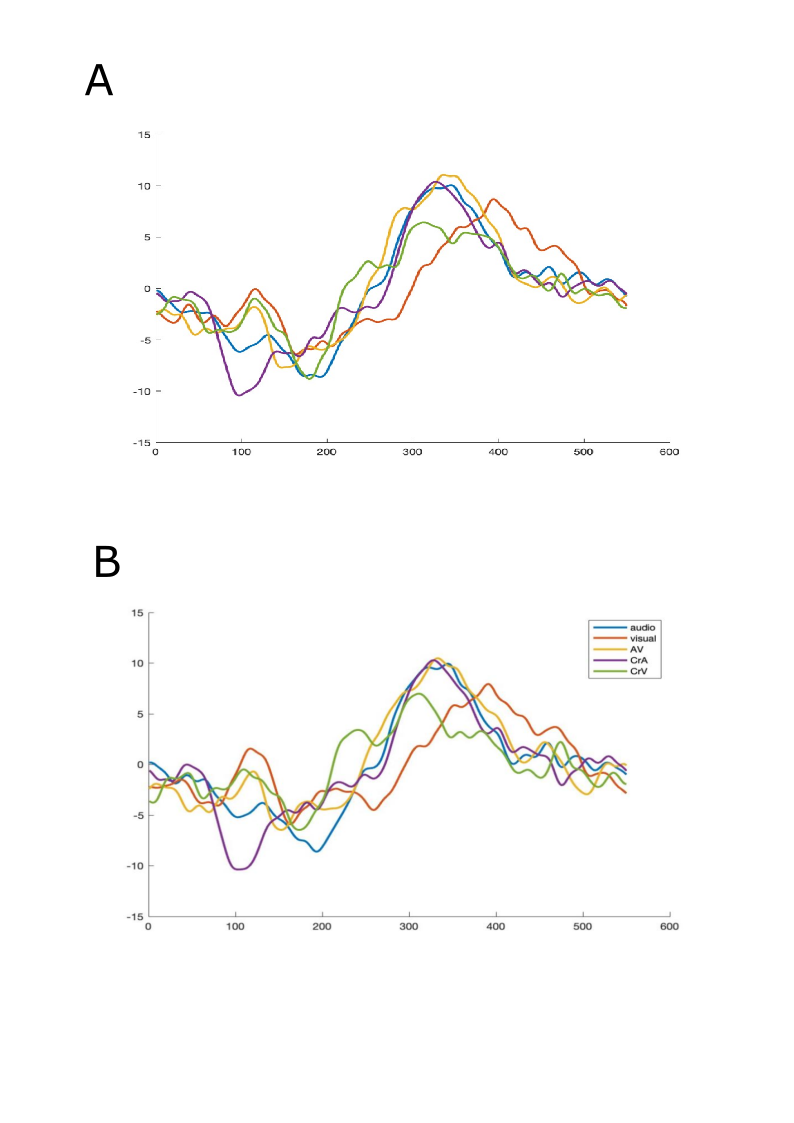

Supplement: Figure 2-1 — A) Common-spatial filter projected on the difference of oddball-standard data (averaged across all participants) for different conditions. B) Condition-specific filter projected on the difference of oddball-standard data (averaged across all participants) for different conditions. Download Figure 2-1, TIF file. [file eneuro-11-ENEURO.0251-23.2024-s003.tif]
