# Supplementary material for: Unsupervised Characterization of Prediction Error Markers in Unisensory and Multisensory Streams Reveal the Spatiotemporal Hierarchy of Cortical Information Processing
Source: eNeuro. 2024 May 2;11(5):ENEURO.0251-23.2024. doi: 10.1523/ENEURO.0251-23.2024 (PMC11069433; doi:10.1523/ENEURO.0251-23.2024)
Supplement: Table 1-2 — The following table lists the signal-to-noise ratio (%) for each participant across all conditions. Please note the SNR was first computed for every channel and then averaged for each participant. Download Table 1-2, DOCX file. [file eneuro-11-ENEURO.0251-23.2024-s005.docx]

**Table 1-2:** The following table lists the signal-to-noise ratio (%) for each participant across all condition. Please note the SNR was first computed for every channel and then averaged for each participant.Please note the SNR was first computed for every channel and then averaged for each participant.

| **Participant #** | ***Audio only*** | ***Visual only*** | ***AV*** | ***Cross-audio*** | ***Cross-visual*** |
| --- | --- | --- | --- | --- | --- |
| 1 | 74.87 | 75.37 | 76.08 | 74.25 | 75.42 |
| 2 | 75.32 | 75.22 | 75.19 | 76.52 | 75.99 |
| 3 | 73.11 | 72.92 | 73.59 | 75.34 | 72.43 |
| 4 | 73.83 | 75.05 | 75.82 | 77.21 | 78.13 |
| 5 | 77.36 | 74.66 | 74.72 | 74.70 | 76.32 |
| 6 | 75.11 | 76.33 | 74.97 | 75.46 | 74.50 |
| 7 | 74.57 | 75.57 | 75.34 | 78.19 | 76.04 |
| 8 | 74.94 | 76.04 | 75.08 | 76.73 | 77.43 |
| 9 | 77.12 | 76.87 | 75.66 | 75.51 | 76.37 |
| 10 | 76.41 | 74.98 | 75.97 | 77.80 | 74.94 |
| 11 | 77.43 | 78.13 | 75.87 | 76.11 | 76.34 |
| 12 | 75.01 | 77.12 | 73.66 | 77.08 | 75.84 |
| 13 | 76.39 | 76.52 | 75.21 | 77.35 | 74.39 |
| 14 | 76.17 | 77.35 | 75.51 | 76.57 | 75.91 |
| 15 | 77.65 | 76.75 | 74.62 | 77.43 | 77.93 |
| 16 | 75.28 | 75.15 | 75.28 | 77.95 | 76.82 |
| 17 | 74.58 | 74.26 | 76.26 | 76.98 | 74.21 |
| 18 | 75.85 | 76.57 | 75.99 | 75.95 | 76.67 |
| 19 | 75.36 | 77.80 | 75.41 | 77.98 | 77.21 |
| 20 | 75.71 | 77.60 | 77.44 | 76.90 | 76.77 |
| 21 | 78.07 | 78.90 | 76.04 | 77.71 | 76.97 |
